# N-Acetylcysteine Attenuates the Development of Renal Fibrosis in Transgenic Mice with Dilated Cardiomyopathy

**DOI:** 10.1038/s41598-017-17927-5

**Published:** 2017-12-18

**Authors:** Beverly Giam, Sanjaya Kuruppu, Po-Yin Chu, A. Ian Smith, Francine Z. Marques, April Fiedler, Duncan Horlock, Helen Kiriazis, Xiao-Jun Du, David M. Kaye, Niwanthi W. Rajapakse

**Affiliations:** 1 Baker Heart and Diabetes Institute, Melbourne, Australia; 2 Central Clinical School, Monash University, Melbourne, Australia; 3 Biomedicine Discovery Institute, Department of Biochemistry & Molecular Biology, Monash University, Melbourne, Australia; 4 Department of Medicine, Monash University, Melbourne, Australia; 5 School of Biomedical Sciences, Faculty of Medicine, The University of Queensland, Brisbane, Australia

## Abstract

Mechanisms underlying the renal pathology in cardiorenal syndrome (CRS) type 2 remain elusive. We hypothesised that renal glutathione deficiency is central to the development of CRS type 2. Glutathione precursor, N-acetylcysteine (NAC;40 mg/kg/day; 8 weeks) or saline were administered to transgenic mice with dilated cardiomyopathy (DCM) and wild-type (WT) controls. Cardiac structure, function and glutathione levels were assessed at the end of this protocol. Renal fibrosis, glutathione content, expression of inflammatory and fibrotic markers, and function were also evaluated. In both genotypes, NAC had minimal effect on cardiac glutathione, structure and function (*P* ≥ 0.20). In NAC treated DCM mice, loss of glomerular filtration rate (GFR), tubulointerstitial and glomerular fibrosis and renal oxidised glutathione levels were attenuated by 38%, 99%, 70% and 52% respectively, compared to saline treated DCM mice (*P* ≤ 0.01). Renal expression of PAI-1 was greater in saline treated DCM mice than in WT mice (*P* < 0.05). Renal PAI-1 expression was less in NAC treated DCM mice than in vehicle treated DCM mice (*P* = 0.03). Renal IL-10 expression was greater in the former cohort compared to the latter (*P* < 0.01). These data indicate that normalisation of renal oxidized glutathione levels attenuates PAI-1 expression and renal inflammation preventing loss of GFR in experimental DCM.

## Introduction

Heart failure (HF) is a clinical syndrome with complex pathophysiology and it is accompanied by significant morbidity and mortality^1,2^. Renal and cardiac dysfunction often occur together and the combination, termed cardiorenal syndrome (CRS), is typically associated with a poorer outcome than that associated with individual organ dysfunction^3^. Chronic renal disease arising in the context of a primary cardiac defect is termed CRS type 2^3^. The renal pathology in CRS type 2 is poorly understood and there are no specific treatment interventions that can reverse or even halt the progression of renal disease in patients with CRS type 2^4^.

Chronic HF is associated with systemic inflammation and augmented levels of circulating pro-inflammatory cytokines such as tumour necrosis factor alpha (TNF-α) and interleukin (IL-)1^5,6^, which can deplete glutathione levels^7,8^. Glutathione deficiency can reduce collagen degradation leading to fibrosis^9,10^. In addition, glutathione deficiency is associated with augmented expression of plasminogen activator inhibitor-1 (PAI-1)^11^ which in turn can facilitate macrophage recruitment and production of pro-inflammatory cytokines^12^ creating a vicious cycle leading to inflammation, glutathione depletion and fibrogenesis.

Glutathione plays a pivotal role in regulating renal function and its deficiency is frequently linked with renal injury. Indeed, in patients with chronic renal failure, changes in glutathione levels and related enzymes have been reported^13,14^. Furthermore, glutathione deficiency *per se* has been demonstrated to induce renal fibrosis and albuminuria in Dahl salt sensitive rats^15^. N-acetylcysteine (NAC), is an antioxidant which serves as a precursor for the formation of glutathione and some evidence of reno-protection has been described in the setting of chronic kidney disease (CKD)^16–18^. However, the association between renal inflammation, glutathione levels and renal function has not been investigated in the setting of CRS type 2. In the present study, we tested that hypothesis that renal glutathione deficiency is central to the progression of renal inflammation and fibrosis leading to loss of renal function in CRS type 2.

There are only a limited number of experimental CRS type 2 models available^19^. Current experimental models of HF, such as that due to myocardial infarction simulated by coronary artery ligation do not appear to develop the level of renal injury and dysfunction observed in patients with CRS type 2^20,21^. Therefore in the present study, we aimed to validate a new mouse model of CRS type 2 and accordingly loss of renal function and inflammation were assessed in a transgenic mouse model of dilated cardiomyopathy (DCM).

## Results

### Renal fibrosis at baseline prior to administration of NAC or saline

**Figure 1:**
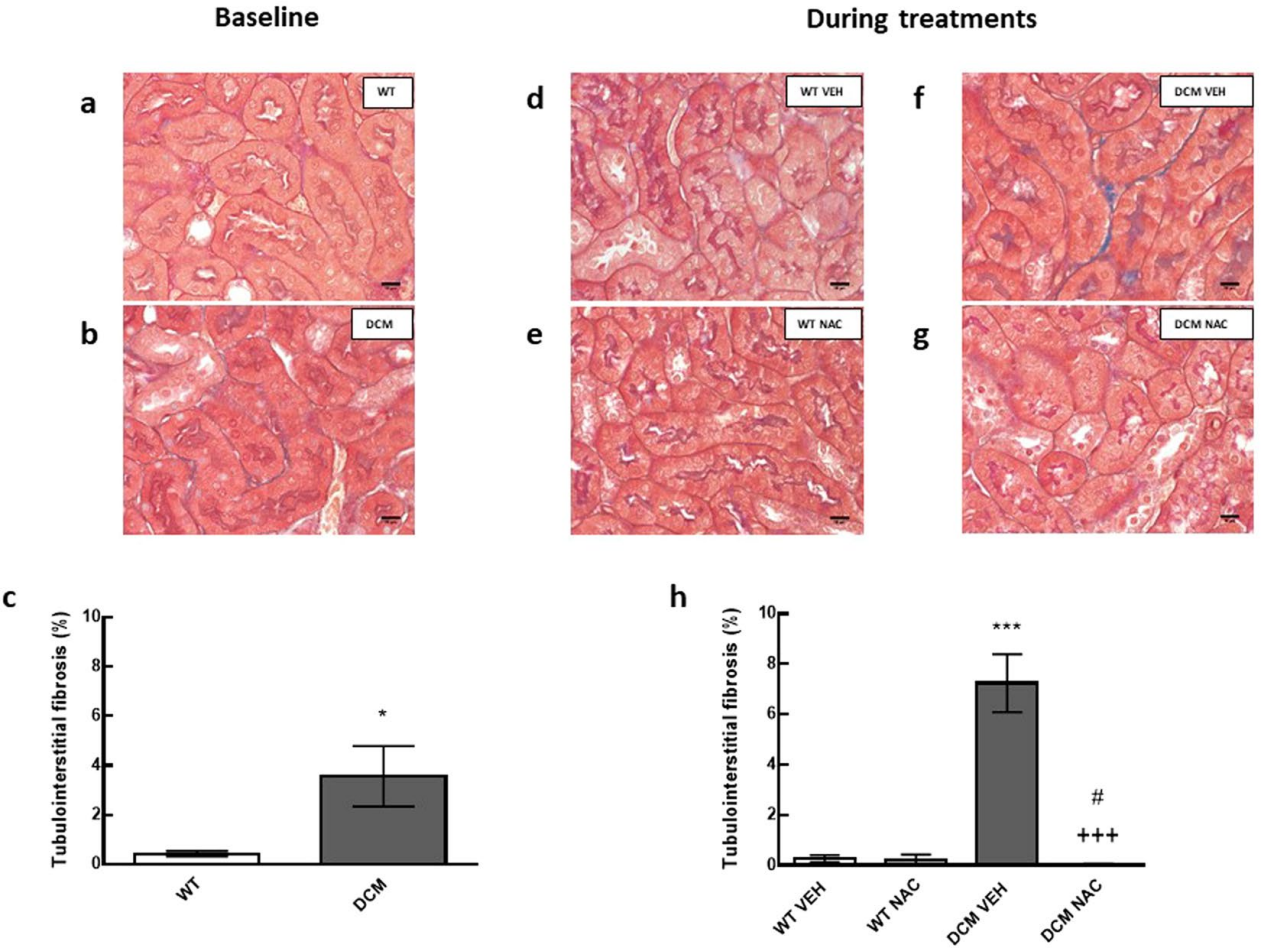
(**a**,**b**) Representative images of tubulointerstitial fibrosis at baseline. Scale bars are 16 µm. Blue staining indicates fibrosis. (**c**) Percentage of tubulointerstitial fibrosis at baseline (*n* = 5). (**d**–**g**) Representative images of tubulointerstitial fibrosis after 8 weeks of NAC or saline treatments. (**h**) Percentage of tubulointerstitial fibrosis after 8 weeks of NAC or saline treatments (*n* = 4–6). Data are mean ± SEM. *P < 0.05, ***P < 0.001 *vs* age matched saline treated WT mice. ^+++^ *P* < 0.001 *vs* age matched vehicle treated DCM mice. ^#^ *P* < 0.05 *vs* DCM mice at baseline. *P* values were derived from a one-way ANOVA followed by Tukey post-hoc test. An unpaired t-test was used to compare baseline fibrosis *vs* fibrosis at the end of NAC treatment in DCM mice. WT = wild type mice, DCM = transgenic mice with dilated cardiomyopathy, VEH = vehicle, NAC = N-acetylcysteine.

**Figure 2:**
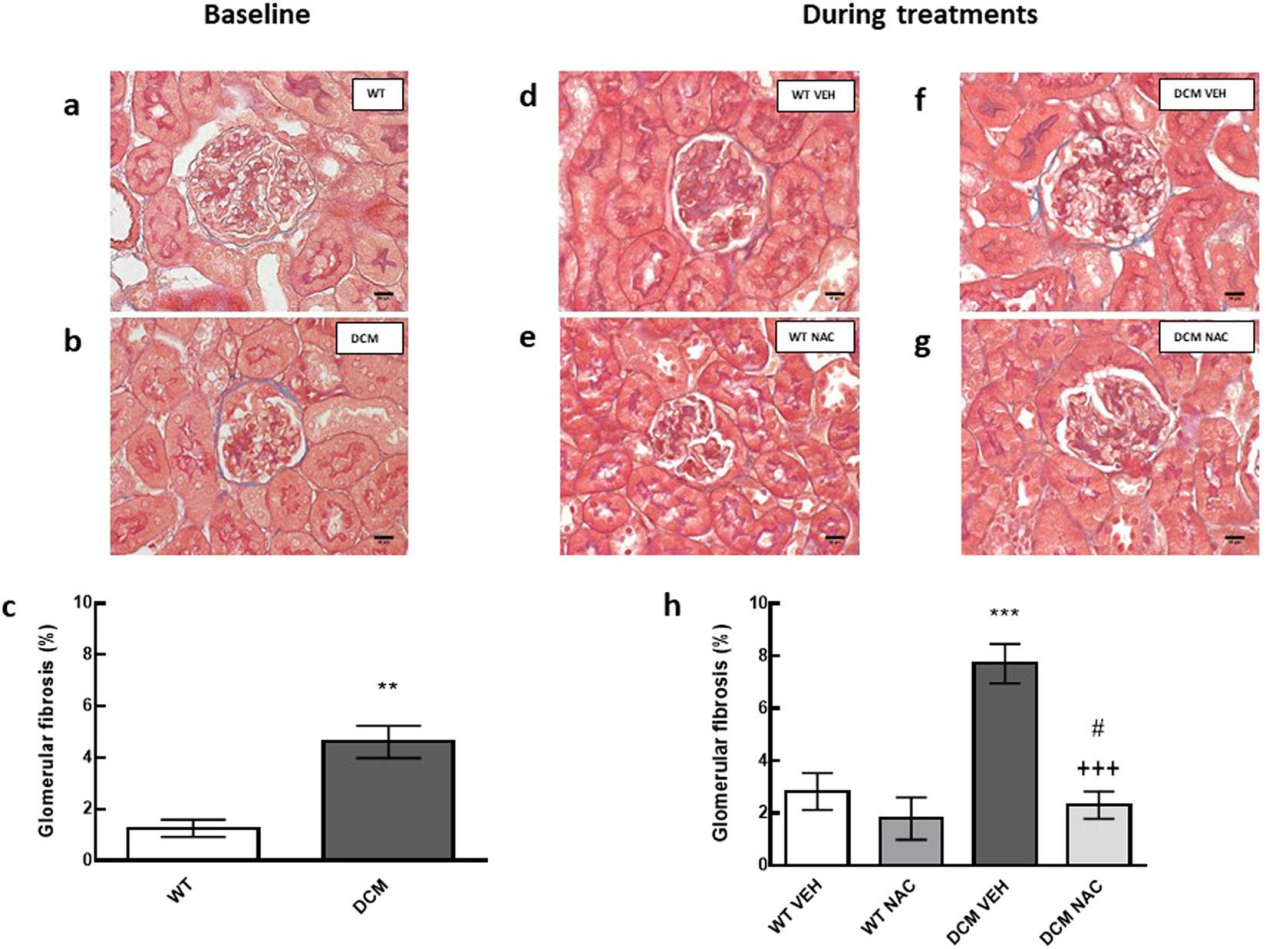
(**a**,**b**) Representative images of glomerular fibrosis at baseline. Scale bars are 16 µm. Blue staining indicates fibrosis. (**c**) Percentage of glomerular fibrosis at baseline (*n* = 5). (**d**–**g**) Representative images of glomerular fibrosis after 8 weeks of NAC or saline treatments. (**h**) Percentage of glomerular fibrosis after 8 weeks of NAC or saline treatments (*n* = 4–6). Data are mean ± SEM. **P < 0.01, ***P < 0.001 *vs* age matched saline treated WT mice. ^+++^ *P* < 0.001 *vs* age matched vehicle treated DCM mice. ^#^ *P* < 0.05 *vs* DCM mice at baseline. *P* values were derived from a one-way ANOVA followed by Tukey post-hoc test. An unpaired t-test was used to compare baseline fibrosis *vs* fibrosis at the end of NAC treatment in DCM mice. Abbreviations are as for Fig. 1.

Tubulointerstitial and glomerular fibrosis were greater in DCM mice (by 88% and 73%, respectively) than in age matched WT mice (*P* ≤ 0.03; Figs 1a–c and 2a–c).

### NAC attenuated the development of renal fibrosis

In DCM mice treated with saline, tubulointerstitial and glomerular fibrosis were greater (by 51% and 40% respectively) than that observed at baseline (*P* ≤ 0.04; Figs 1d–h and 2d–h). In contrast, in NAC treated DCM mice, glomerular fibrosis, and in particular, tubulointerstitial fibrosis, were less compared to respective levels of fibrosis observed at baseline (*P* ≤ 0.04; Figs 1 and 2). Indeed, tubulointerstitial and glomerular fibrosis were 99% and 70% less respectively, in NAC treated DCM mice, when compared to saline treated DCM mice (*P* ≤ 0.001; Figs 1d–h and 2d–h).

### NAC prevented the loss of glomerular filtration rate

**Figure 3:**
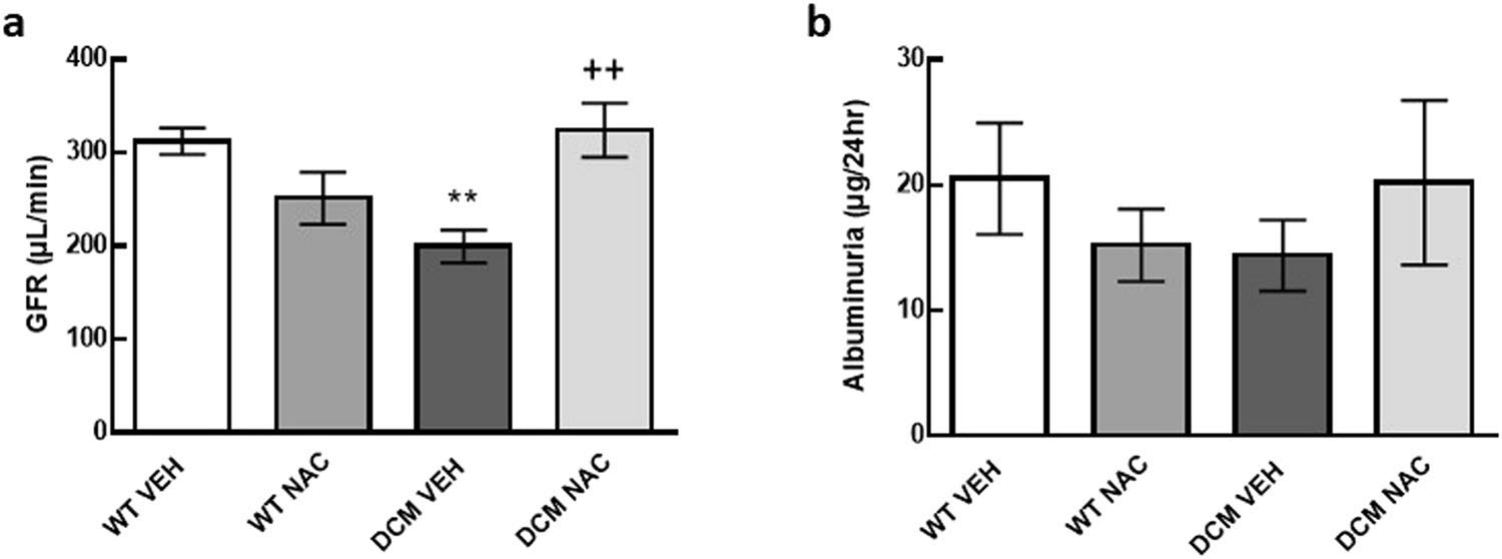
(**a**) Glomerular filtration rate (as measured by creatinine clearance) and (**b**) albuminuria in WT and DCM mice administered NAC or saline (*n* = 7–9). Data are mean ± SEM. **P < 0.01 *vs* saline treated WT mice. ^++^ *P* < 0.01 *vs* saline treated DCM mice. *P* values were derived from a one-way ANOVA followed by Tukey post-hoc test. GFR = glomerular filtration rate. Other abbreviations are as for Fig. 1.

GFR was 36% less in saline treated DCM mice than in saline treated WT mice (*P* = 0.01; Fig. 3a). Importantly, GFR was 38% greater in NAC treated DCM mice than in saline treated DCM mice (*P* = 0.003; Fig. 3a). Indeed, after 8 weeks of NAC treatment, GFR in DCM mice was comparable to that of age matched WT mice (*P* = 0.99; Fig. 3a).

### NAC had minimal effect on albuminuria

Albuminuria was not significantly different between groups (*P* ≥ 0.60; Fig. 3b).

### Renal glutathione content at baseline

**Figure 4:**
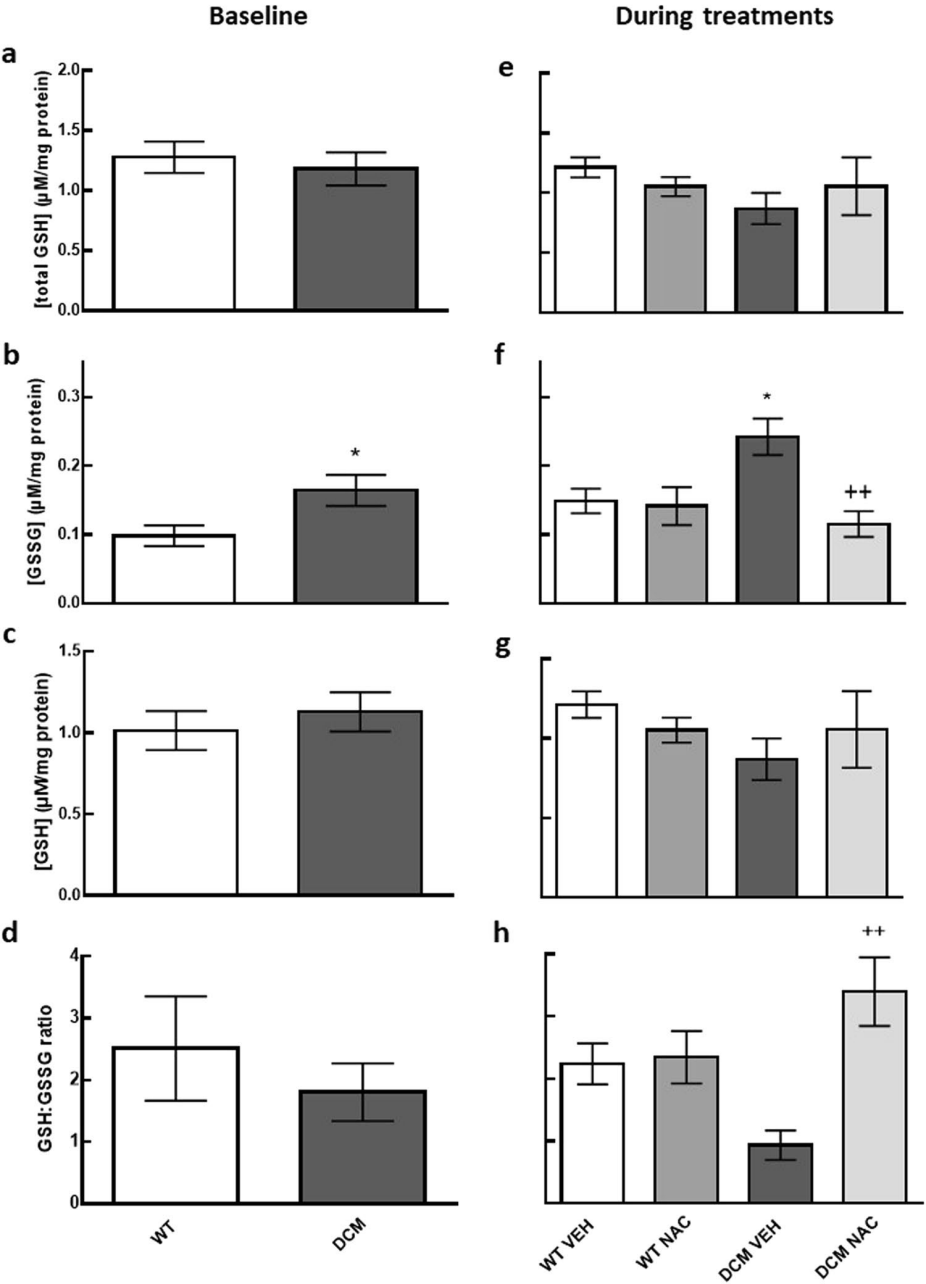
Levels of glutathione (**a**–**d**) at baseline (*n* = 4–6) and (**e**–**h**) after 8 weeks of NAC or saline treatments (*n* = 6). Data are mean ± SEM. *P ≤ 0.05 *vs* age matched WT mice. ^++^ *P* < 0.01 *vs* age matched saline treated DCM mice. *P* values were derived from a one-way ANOVA followed by Tukey post-hoc test. Total GSH = total glutathione content, GSSG = oxidised glutathione content, GSH = reduced glutathione content, GSH:GSSG = reduced glutathione: oxidised glutathione ratio. Other abbreviations are as for Fig. 1.

Oxidised glutathione levels in the kidney were 40% greater in DCM mice than in WT mice (*P* = 0.05; Fig. 4b).

### NAC normalised renal glutathione content

Oxidised glutathione levels were 39% greater in saline treated DCM mice compared to saline treated WT mice (*P* = 0.04; Fig. 4f). In contrast, levels of oxidised renal glutathione were 52% less in NAC treated DCM mice compared to saline treated DCM mice (*P* = 0.005; Fig. 4f). The ratio of reduced glutathione to oxidised glutathione was 73% greater in NAC treated DCM mice compared to saline treated DCM mice (*P* = 0.002; Fig. 4h).

### Assessment of collagen content in the renal cortex

**Figure 5:**
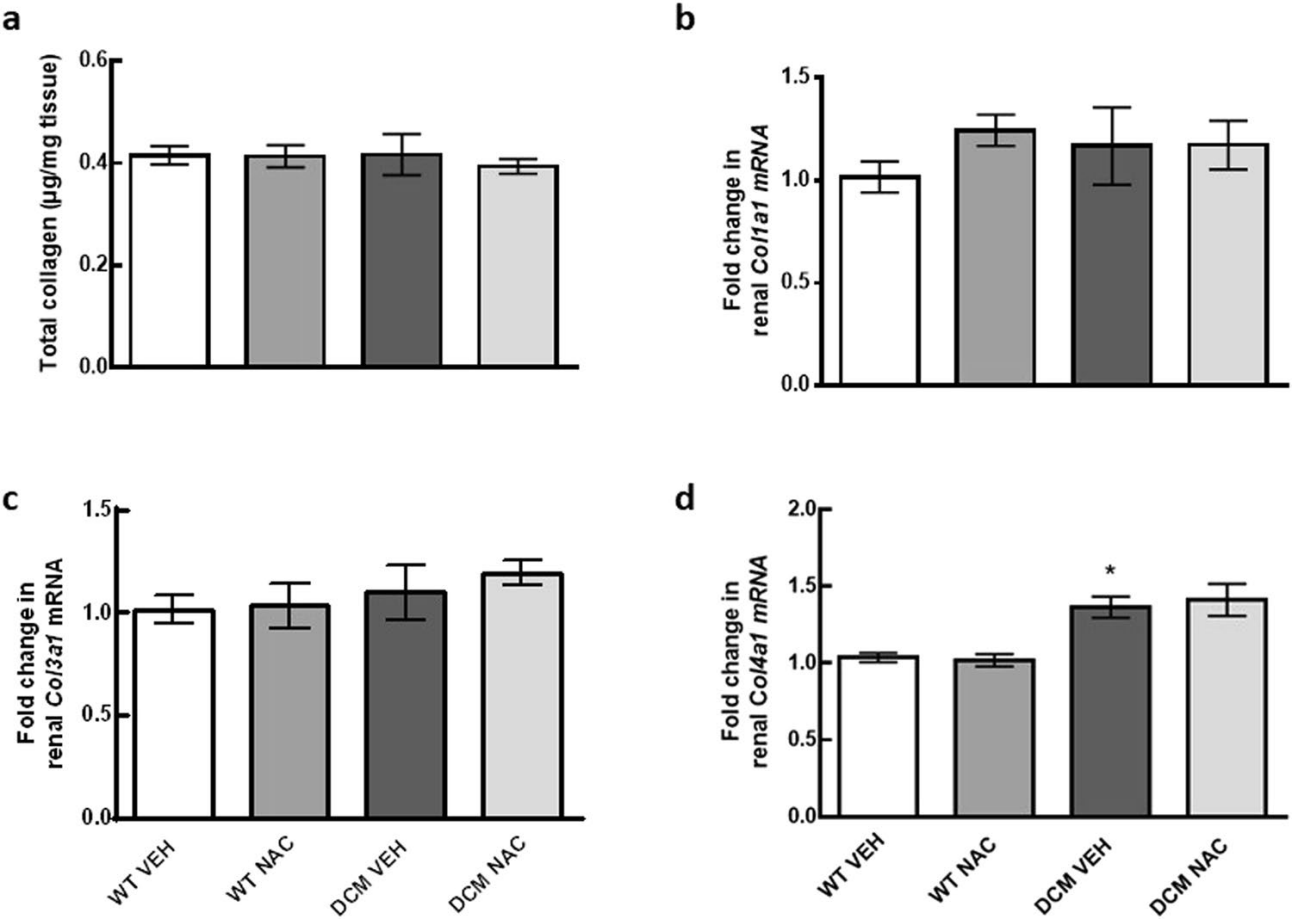
(**a**–**d**) Expression of total collagen, collagen types I, III and IV in renal cortical tissues of WT and DCM mice administered NAC or saline (*n* = 4–8). Data are mean ± SEM. *P < 0.05, **P < 0.01 *vs* saline treated WT mice. *P* values were derived from a one-way ANOVA followed by Tukey post-hoc test. Col1a1 = collagen type I, Col3a1 = collagen type III, Col4a1 = collagen type IV. Other abbreviations are as for Fig. 1.

Total collagen content including procollagen, mature collagen and degraded collagen proteins as measured by the hydroxypoline assay was not significantly different between groups (*P* ≥ 0.93; Fig. 5a).

Renal mRNA expression of collagen type I (*Col1a1*) and collagen type III (*Col3a1*) was not significantly different between groups (*P* ≥ 0.43; Fig. 5b,c) but mRNA expression of collagen type IV (*Col4a1*) was greater in saline treated DCM mice compared to saline treated WT mice (*P* = 0.02; Fig. 5d).

### Expression of pro-inflammatory cytokines in the kidney

**Figure 6:**
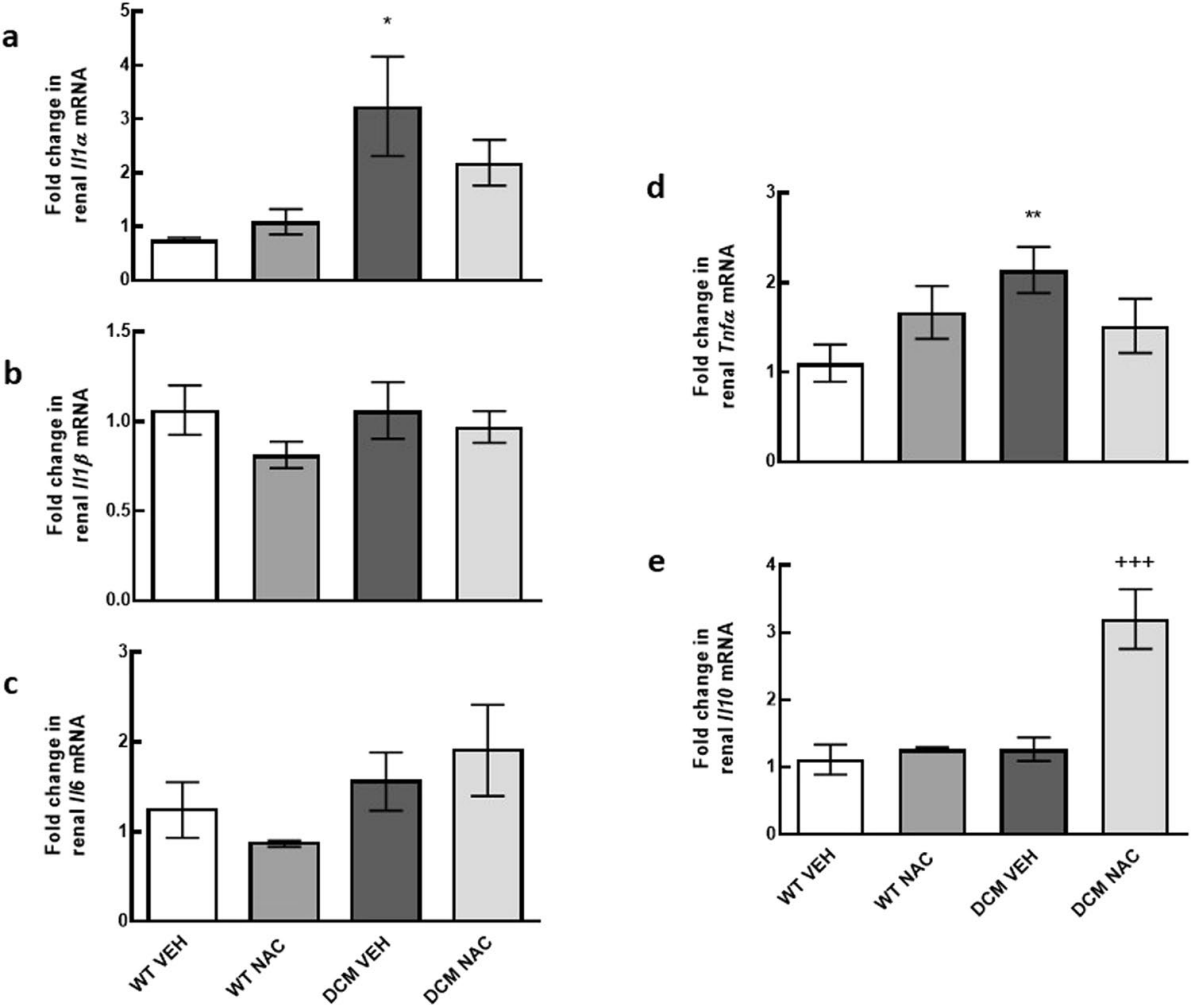
(**a**–**e**) Markers associated with inflammation induced renal fibrosis. mRNA expression of *Il1a*, *Il1b*, *Il6*, *Tnfα* and *Il10* in renal cortical tissues of WT and DCM mice administered NAC or saline (*n* = 6–8). Data are mean ± SEM. *P < 0.05, **P < 0.01 *vs* saline treated WT mice. ^+++^ *P* < 0.001 *vs* saline treated DCM mice. *Il1a* = interleukin 1 alpha, *Il1b* = interleukin 1 beta, *Il6* = interleukin 6, *Tnfα* = tumor necrosis factor alpha, *Il10* = interleukin 10. Other abbreviations are as for Fig. 1.

mRNA expression of *Il1a* and *Tnfα* were 74% and 49% greater respectively, in saline treated DCM mice compared to saline treated WT mice (*P* ≤ 0.02; Fig. 6a and d). NAC had minimal effect on *Il1a* and *Tnfα* mRNA expression in DCM mice (*P* ≥ 0.42; Fig. 6a and d). mRNA expression levels of *Il1b* and *Il6* were not significantly different between groups (*P* ≥ 0.17; Fig. 6b,c).

### Expression of anti-inflammatory cytokines in the kidney

mRNA expression of *Il10* was 74% greater in NAC treated DCM mice compared to saline treated DCM mice (*P* = 0.0003; Fig. 6e).

**Figure 7:**
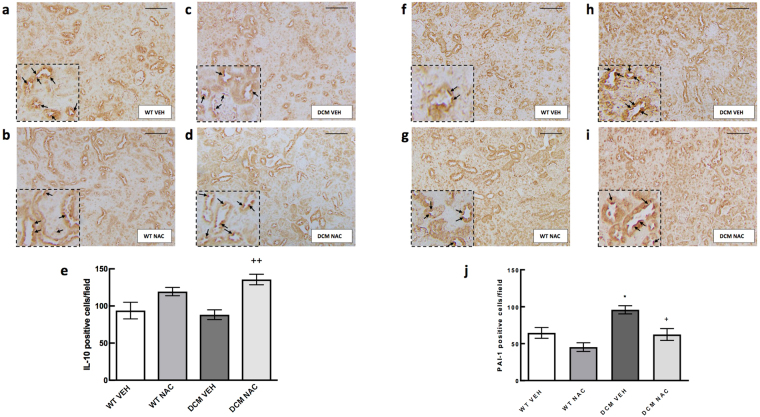
Representative images of renal expression of (**a**–**d**) IL-10 and (**f**–**i**) PAI-1 after 8 weeks of NAC or saline treatments. Magnified section of each representative image (outlined with dotted lines) has been included to provide clear representation of positive cells. Here, black arrows were used to indicate positive cells. Scale bars are 100 µm. Quantification of the mean immunoexpression of (**e**) IL-10 and (**j**) PAI-1 in each group after 8 weeks of NAC or saline treatments (*n* = 3–4). Data are mean ± SEM. *P < 0.05 *vs* saline treated WT mice. ^+^ *P* < 0.05, ^++^ *P* < 0.01 *vs* vehicle treated DCM mice. *P* values were derived from a one-way ANOVA followed by Tukey post-hoc test. IL-10 = interleukin 10, PAI-1 = plasminogen activator inhibitor-1. Other abbreviations are as for Fig. 1.

Renal cortical protein expression of IL-10 was not significantly different between DCM mice and WT mice (*P* ≥ 0.95; Fig. 7e). Of interest, protein expression of IL-10 was 35% greater in DCM mice administered NAC compared to those administered saline vehicle (*P* = 0.006; Fig. 7e).

### Expression of PAI-1 in the kidney

**Figure 8:**
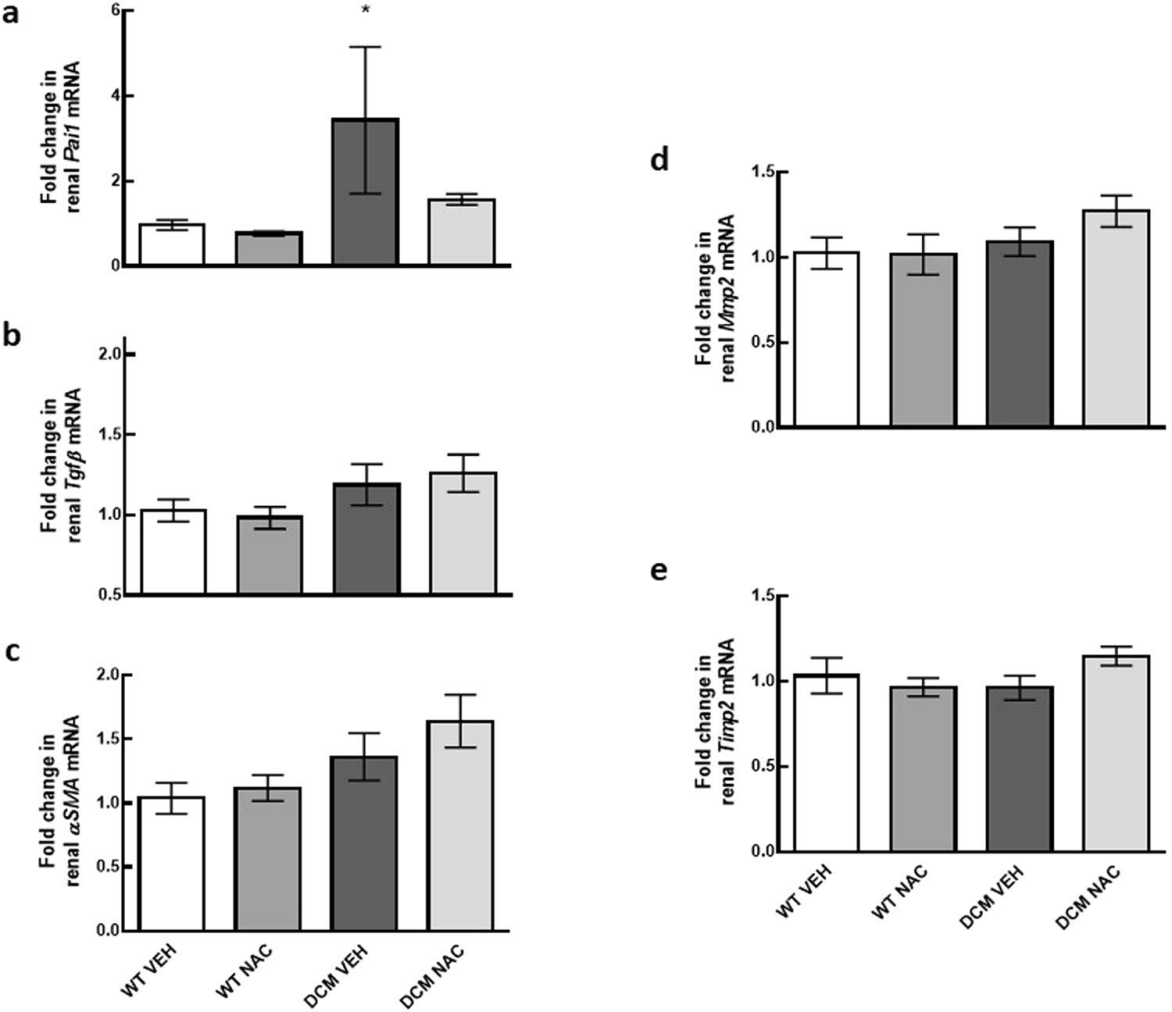
(**a**–**e**) Markers associated with TGF pathway induced renal fibrosis. mRNA expression of *Pai1*, *Tgfβ*, *αSMA*, *Mmp2* and *Timp2* in renal cortical tissues of WT and DCM mice administered NAC or saline (*n* = 5–8). Data are mean ± SEM. *P < 0.05 *vs* saline treated WT mice. *P* values were derived from a one-way ANOVA followed by Tukey post-hoc test. *Pai1* = plasminogen activator inhibitor-1, *Tgfβ* = transforming growth factor beta, *αSMA* = alpha smooth muscle actin, *Mmp2* = matrix metalloproteinase-2, *Timp2* = tissue inhibitor of metalloproteinase 2. Other abbreviations are as for Fig. 1.

Expression of *Pai1* mRNA was 72% greater in saline treated DCM mice compared to saline treated WT mice (*P* = 0.04; Fig. 8a). NAC had minimal effect on *Pai1* mRNA expression (*P* ≥ 0.25; Fig. 8a).

Renal protein expression of PAI-1 was 33% greater in vehicle treated DCM mice compared to vehicle treated WT mice (*P* = 0.03; Fig. 7j). Importantly, protein expression of PAI-1 was 35% less in NAC treated DCM mice than in vehicle treated DCM mice (*P* = 0.03; Fig. 7j).

### Expression of fibrotic markers relating to TGF-β pathway

mRNA expression of transforming growth factor beta (*Tgfβ*), alpha smooth muscle actin (*αSMA*), matrix metalloproteinase-2 (*Mmp2*) and tissue inhibitor of metalloproteinase 2 (*Timp2*) were not significantly different between groups (*P* ≥ 0.09; Fig. 8b–e).

### Baseline cardiac structure and function prior to administration of NAC or saline

The thickness of the interventricular septum during diastole and systole were 35% and 44% less respectively, in DCM mice than in WT mice (*P* < 0.001; Supplementary Table S2). The thickness of the left ventricular posterior wall during systole was 25% less in DCM mice than in WT mice (*P* < 0.001; Supplementary Table S2). Left ventricular end-systolic dimension was 24% greater in DCM mice than in WT mice (*P* < 0.001; Supplementary Table S2). Other indices of cardiac structure did not significantly differ between DCM mice and WT mice (Supplementary Table S2).

Fractional shortening was 44% less in DCM mice than in age matched WT mice (*P* < 0.001; Supplementary Table S1).

### NAC had minimal effect on cardiac structure and function

The thickness of the interventricular septum during diastole and systole were 32% and 39% less respectively, in saline treated DCM mice compared to saline treated WT mice (*P* < 0.001; Supplementary Table S3). The thickness of the left ventricular posterior wall during diastole and systole was 17% and 35% less respectively, in saline treated DCM mice compared to saline treated WT mice (*P* ≤ 0.004; Supplementary Table S3). Left ventricular end-systolic dimension was 33% greater in saline treated DCM mice compared to saline treated WT mice (*P* < 0.001; Supplementary Table S3). Other indices of cardiac structure did not significantly differ between DCM and WT mice (Supplementary Table S3). NAC had minimal effect on all echocardiographic measures of cardiac structure and function (Supplementary Table S1 and Supplementary Table S3).

In DCM mice treated with saline, fractional shortening and d*P*/d*t*_min_ were 57% and 41% less respectively, than in WT mice treated with saline (*P* ≤ 0.002; Supplementary Table S1 and Supplementary Fig. S1g). Tau was 40% greater in saline treated DCM mice compared to saline treated WT mice (*P* < 0.01; Supplementary Fig. S1h). Treatment with NAC had minimal effect on all measures of cardiac function (*P* ≥ 0.32; Supplementary Table S1 and Supplementary Fig. S1f–h).

### NAC had minimal effect on aortic and ventricular pressure

Arterial and left ventricular pressures were measured during the 8^th^ week of NAC or saline treatment when mice were 26 weeks old. Heart rate was not significantly different between saline treated DCM mice and saline treated WT mice (*P* ≥ 0.16; Supplementary Fig. S1a). Systolic and diastolic pressure were 20% and 23% less respectively in saline treated DCM mice compared to saline treated WT mice (*P* < 0.01; Supplementary Fig. S1b,c). Left ventricular systolic pressure was 23% less in saline treated DCM mice compared to saline treated WT mice (*P* < 0.001; Supplementary Fig. S1d). Left ventricular end-diastolic pressure was 59% greater in saline treated DCM mice compared to saline treated WT mice (*P* < 0.001; Supplementary Fig. S1e). Treatment with NAC had minimal effect on arterial and left ventricular pressure (*P* ≥ 0.67; Supplementary Fig. S1b–e).

### NAC had minimal effect on cardiac glutathione content

Cardiac oxidised glutathione levels were 27% greater in saline treated DCM mice compared to saline treated WT mice (*P* = 0.009; Supplementary Fig. S2). Treatment with NAC had minimal effect on oxidised glutathione content in DCM mice (*P* = 0.94; Supplementary Fig. S2).

### NAC had no effect on body weight and lung weight

Body weight was not significantly different between groups (*P* ≥ 0.21; Supplementary Table S4). Lung weight was 22% greater in DCM mice compared to WT mice (*P* = 0.004; Supplementary Table S4). Treatment with NAC had minimal effect on body weight and lung weight (*P* ≥ 0.32; Supplementary Table S4).

## Discussion

The present study demonstrates several key findings of relevance to the pathogenesis and treatment of CRS type 2. First, we validated a new experimental model of CRS type 2. We found that experimental DCM arising from cardiac specific overexpression of *Mst1* was associated with renal fibrosis, inflammation and loss of GFR. Second, we found that NAC treatment normalised renal glutathione content, completely prevented the development of tubulointerstitial fibrosis and substantially reduced the development of glomerular fibrosis in DCM mice. Consistent with this, we also found that loss of GFR was prevented in DCM mice treated with NAC but not saline vehicle. Third, we found that expression of anti-inflammatory cytokine IL-10 was greater while PAI-1 expression was less in DCM mice treated with NAC than those treated with saline vehicle. These data indicate that renal inflammation coupled with augmented expression of pro-fibrotic protein PAI-1 underpin development of renal fibrosis and loss of GFR in experimental DCM.

The reduction in GFR observed in the DCM model is associated with augmented oxidised glutathione levels, renal inflammation and fibrosis. We found that NAC attenuated the development of renal fibrosis and prevented the loss of GFR in DCM mice. Even small amounts of renal fibrosis can lead to significant loss of renal function. For example, minimal levels of tubulointerstitial fibrosis (3%) was associated with marked reductions in GFR (39%) in a rat model of diabetic nephropathy^22^. Our data are in agreement with previous findings which indicate that regression of renal fibrosis is associated with restoration of renal function^22,23^. Blood pressure and fractional shortening were less in DCM mice and each has been correlated with renal dysfunction in HF patients^24,25^. However, in the current study, NAC maintained GFR in DCM mice in the absence of an improvement in blood pressure or fractional shortening. Preservation of renal function is of utmost significance in the setting of HF as impaired renal function precludes treatment interventions for HF patients. Our present data indicate that NAC can potentially rescue renal function in HF patients which merits further investigation in the future.

Findings from the present study strongly support our hypothesis that glutathione deficiency within the kidney is central to the development of renal dysfunction in DCM. We found that glutathione precursor NAC normalised renal oxidised glutathione levels together with GFR in DCM mice. Of particular interest, the levels of tubulointerstitial and glomerular fibrosis in NAC treated DCM mice were 99% and 70% less respectively, compared to saline treated DCM mice. Together, these data indicate that normalisation of glutathione levels within the kidney can preserve renal function by preventing the development of renal fibrosis. It has previously been demonstrated that there is a positive correlation between creatinine clearance and plasma glutathione peroxidase activity in patients with CKD^14^. There is also direct evidence that increasing oxidised glutathione content in the kidney via administration of methylglyoxal can induce renal tubulointerstitial fibrosis and albuminuria in Dahl salt-sensitive rats^15^. Furthermore, it has been demonstrated that depletion of glutathione leads to fibrogenesis in cultured renal epithelial cells and in cultured mouse embryonic fibroblasts^11,26^. Subsequent replenishment of glutathione in cultured mouse embryonic fibroblasts increased collagen degradation rate^11^. Together, these data indicate that glutathione can attenuate development of renal fibrosis potentially via accelerating the rate of collagen degradation. In line with this, we found that expression of collagen type I protein as measured by Masson’s trichrome staining was greater in saline treated DCM mice compared to WT mice, and this was significantly less in NAC treated DCM mice compared to saline treated DCM mice. However, mRNA expression of collagen type I and total collagen protein content were not significantly different between NAC and saline treated DCM mice despite the augmented expression of collagen type I protein. This suggests that the renal anti-fibrotic effects of NAC may be mediated via augmented collagen type I degradation but not via reduced collagen type I synthesis.

The pathogenic role of inflammation is well defined in the context of chronic HF and to a lesser extent in the setting of CRS type 2. In the current study, we investigated whether reduced renal glutathione levels in CRS type 2 was associated with renal inflammation. We found that expression of pro-inflammatory cytokines *Tnfα* and *Il1a* were augmented in DCM mice. NAC, despite normalising glutathione levels and exerting reno-protective effects in DCM mice, had minimal effect on the expression of these cytokines, suggesting that inflammation occurs upstream to glutathione depletion (Supplementary Fig. S3). In support of this notion, it has been demonstrated that TNF-α can reduce total glutathione levels and increase oxidised levels of glutathione in bovine pulmonary microvascular endothelial cells^8^. Likewise, IL-1 is demonstrated to increase oxidised glutathione levels in human fibroblasts^7^. Together, these findings suggest that augmented levels of TNF-α and IL-1 deplete renal glutathione levels in CRS type 2. Furthermore, TNF-α can increase PAI-1 expression^12^ potentially setting into motion a cascade of events ultimately leading to inflammation and fibrosis. Augmented expression of PAI-1 inhibits the degradation of collagen^11^, and is associated with renal macrophage recruitment^27^ which in turn can produce a range of pro-inflammatory markers including TNF-α and IL-1^28,29^. This can create a vicious cycle leading to inflammation, reduced glutathione content and fibrogenesis. Importantly, our present data indicate that restoration of glutathione via provision of NAC can interrupt this cycle and rescue renal function in the setting of CRS type 2 (Supplementary Fig. S3). We have found that both mRNA and protein expression of PAI-1 was greater in saline treated DCM mice compared to saline treated WT counterparts. PAI-1 expression was less in NAC treated DCM mice than in saline treated DCM mice. In addition, we found that renal mRNA and protein expression of anti-inflammatory cytokine IL-10 was augmented in DCM mice treated with NAC. Consistent with this, it has been demonstrated that NAC can increase IL-10 in peripheral blood mononuclear cells^30^. IL-10 has been shown to reduce renal fibrosis by reducing macrophage infiltration and apoptosis^31,32^. It is also well established that IL-10 can independently downregulate the expression of pro-inflammatory cytokines IL-1 and TNF-α^33^. Together, our data indicate that two different mechanisms are likely to underpin the anti-fibrotic effects of NAC. First by reducing renal inflammation, and second by inhibiting the expression of PAI-1, both of which can prevent the development of renal fibrosis and preserve renal function in CRS type 2 (Supplementary Fig. S3).

Treatment with NAC had no effect on cardiac oxidised glutathione content in DCM mice, which may explain the lack of effect of NAC on cardiac structure and function. Alternatively, NAC may have failed to mitigate the deleterious effects of cardiac specific overexpression of the *Mst1* transgene in these mice. Cardiac specific overexpression of the *Mst1* transgene in mice leads to apoptosis of cardiomyocytes, cardiac fibrosis and DCM^34^. While NAC did not exert any cardio-protective effects in our experimental model, our data indicate that NAC restored renal glutathione levels and rescued renal function in the setting of established DCM.

There are only a few experimental models of CRS type 2^35^, most of which were created by myocardial infarction together with either uni-nephrectomy^36^ or sub-nephrectomy^37^. However, none of these models closely mimic the renal pathology in CRS type 2 patients^19^. These models displayed variable renal pathology and loss of GFR was not substantial compared to that observed in patients^38–41^. Our present data indicate that DCM mice have significant loss of renal function together with progressive development of renal fibrosis, which are salient features of CRS type 2^42^. Thus, this model should be useful to study molecular mechanisms underlying CRS type 2 as well as to investigate new treatment interventions for this disease state.

In summary, our data indicate that CRS type 2 is associated with renal inflammation, glutathione deficiency and fibrosis. Restoration of renal glutathione levels via administration of NAC led to attenuation of renal fibrosis, augmented expression of anti-inflammatory cytokine IL-10, inhibition of PAI-1 expression and preservation of renal function in experimental DCM. Impaired renal function can increase the morbidity and mortality in HF patients and accordingly, it is of great interest to further investigate whether treatment interventions that normalise renal glutathione levels can rescue renal function in CRS type 2.

## Methods

### Ethical approval

The current study was approved by the Alfred Medical Research and Education Precinct Animal Ethics Committee and all protocols were conducted in accordance with the Australian Code for Care and Use of Animals for Scientific Purposes (8^th^ edition, 2013).

### Transgenic mouse model with DCM

Sixteen male mice with DCM and eighteen littermate controls were used. In DCM mice, the mammalian sterile 20-like kinase-1 (*Mst1*) transgene, which is under the control of α-myosin heavy chain promoter, is overexpressed in a cardiac specific manner^34^. *Mst1* is a well-established mediator of apoptotic cell signalling^34^. This causes cardiomyocytes to undergo apoptosis, leading to DCM and ultimately HF^34^. It has previously been demonstrated that DCM mice develop evidence of cardiac dysfunction as reflected by 33% reduction in left ventricular ejection fraction from 10 weeks of age^34^.

### Protocol 1: Assessment of renal fibrosis and glutathione levels at baseline

Eighteen week old male DCM (*n* = 17) and WT mice (*n* = 16) were humanely killed via CO_2_ asphyxiation and heart and kidneys were collected for later analyses of fibrosis and glutathione content.

### Protocol 2: Assessment of creatinine clearance, albuminuria, fibrosis and renal glutathione levels following administration of NAC or saline vehicle

Eighteen week old male DCM mice (*n* = 16) and their WT littermates (*n* = 18) were used in this protocol. Cardiac structure and function were assessed via echocardiography as previously described by us^43^. Forty eight hours later, mice were randomly allocated to receive either NAC (40 mg/kg/day; Sigma-Aldrich, Sydney, NSW, Australia; *n* = 16) or saline vehicle (0.25 µl/h; *n* = 18) for 8 weeks via subcutaneously implanted osmotic minipumps (Alzet Model 2004; Alzet Corporation, Cupertino, CA, USA). The concentration of NAC was chosen based on a previous study conducted by us, where treatment with NAC at 40 mg/kg/day attenuated cardiac fibrosis and remodelling in DCM mice^44^. Minipumps were implanted as previously described by us^44^. Mice were placed in metabolic cages during the eighth week of NAC or saline administration to collect 24-h urine samples for later analyses of albuminuria and creatinine clearance. Echocardiography was also repeated during the eighth week of NAC or saline administration. Two days after performing echocardiography, cardiac catheterisation was performed to determine arterial and left ventricular blood pressure as previously described by us^45^. Mice were then humanely killed by rapid excision of the heart while under deep isoflurane anaesthesia. Heart and kidneys were collected for later analyses of fibrosis, glutathione content and expression of inflammatory markers.

### Estimation of glomerular filtration rate via creatinine clearance

Creatinine concentration in plasma and urine was assessed as previously described^46^. Briefly, acetonitrile (500 µl) was added to urine (20 µl) and plasma (20 µl) samples and mixed by vortexing. Samples were centrifuged (12,000 g for 10 min at 4 °C), the supernatant was collected and evaporated in a Speed-Vac (Labconco, Kansas city, MO, USA). The residue was reconstituted in sodium acetate buffer (5 mM, pH 5.1), and centrifuged for 5 min at 12,000 g. Creatinine levels in the supernatant were determined using a Shodex IEC SP-825 column (8 × 75 mm,) fixed to an Agilent 1100 high-performance liquid chromatography system. Elution was performed using the following conditions of buffer B (5 mM Sodium acetate, and 5 M Sodium chloride, pH 5.1): 20–60% over 20 min. The specific peak corresponding to creatinine was confirmed by comparison with the authentic standard. Manual integration was then used to calculate the area of peak corresponding to creatinine. The amount of creatinine was calculated from a standard curve of known creatinine concentrations. Creatinine clearance was estimated using the following formula: (Urine creatinine concentration X Urine flow rate)/Plasma creatinine concentration.

### Assessment of albuminuria

Albuminuria was measured using a Mouse Albumin ELISA quantitation kit according to the manufacturer’s protocol (E90-134; Bethyl Laboratories Inc; Montgomery, TX, USA)^47^.

### Histological assessment of renal fibrosis

Four-micron thick paraffin sections of kidneys were used for the histological analysis of fibrosis. Each section was stained with Masson’s trichrome and ten random fields were imaged in the renal cortical region using Olympus BH2 microscope (x40 magnification; Olympus, Tokyo, Japan). More specifically, up to three glomeruli per field were imaged for analysis of glomerular fibrosis. To assess glomerular fibrosis, each glomerular capsule was traced using the Image Pro-Plus software (Adept Electronic Solutions Pty Ltd, Moorabbin, Australia) as previously described^48^. Extent of fibrosis was expressed as a percentage of total area of interest.

### Quantification of glutathione levels

Renal cortical and cardiac ventricular sections were homogenized in cold buffer using TissueRupter (Qiagen; Melbourne, Australia), then centrifuged for 15 min (10,000 g at 4 °C). Protein content in the supernatant was quantified using a protein assay (Bio-Rad Laboratories, Hercules, CA, USA). The remaining supernatant was then deproteinated and levels of total glutathione as well as oxidised glutathione were measured using a colorimetric assay kit (703002; Cayman Chemical, Ann Arbor, MI, USA)^49^.

### Real time PCR (qPCR)

RNA was extracted using TRIzol (Thermo Fisher Scientific, Massachusetts, USA). First-strand complementary synthesis reaction was performed using the High Capacity cDNA Reverse Transcription Kit (Thermo Fisher Scientific, Massachusetts, USA). Renal cortical mRNA expression of *Col1a1*, *Col3a1*, *Col4a1*, *Il1a*, *Ilb*, *Il6*, *Il10*, *Tnfα*, *Pai1*, *Tgfβ*, *αSMA*, *Mmp2* and *Timp2* were quantified by qPCR as previously described^50^. SYBR Green PCR master mix was used for amplification reactions in a QuantStudio 7 Flex Real-Time PCR system (both from Thermo Fisher Scientific, Massachusetts, USA). All samples were run in duplicate using the QuantStudio 7 Flex Real-Time PCR system (Thermo Fisher Scientific; Massachusetts, USA). Samples were run in duplicates. The specificity of the qPCR was ensured through melting curve analysis and electrophoresis in agarose gels. The glyceraldehyde 3-phosphate dehydrogenase (*Gapdh*) gene was used as reference transcript. Significance was assessed by 2^−ΔΔCT^. The following primers were used for *Gapdh* (forward: GGGGCTCTCTGCTCCTCCCTG and reverse: ACGGCCAAATCCGTTCACACC), *Col1a1* (forward: GATTGAGAACATCCGCAGCC and reverse: TACTCTCCGCTCTTCCAGTCA), *Col3a1* (forward: ACACGCAAGGCAATGAGACT and reverse: AAGCAAACAGGGCCAATGTC), *Col4a1* (forward: GGCTCTCCGGGTTCAATAGG and reverse: GCCGATGTCTCCACGACTAC), *Il1a* (forward: CGCTTGAGTCGGCAAAGAAATC and reverse: GAGAGAGATGGTCAATGGCAGA), *Ilb* (forward: TGCCACCTTTTGACAGTGATG and reverse: ATGTGCTGCTGCGAGATTTG), *Il6* (forward: TCGTGGAAATGAGAAAAGAGTTGTG and reverse: TCCAGTTTGGTAGCATCCATCAT), *Il10* (forward: TAATAAGCTCCAAGACCAAGGTG and reverse: TCCAGCAGACTCAATACACACT), *Tnfα* (forward: ATCGGTCCCCAAGATGA and reverse: TGGTGGTTTGTGAGTGTGAGG), *Pai1* (forward: TCTCCAATTACTGGGTGAGTCAG and reverse: GCAGCCGGAAATGACACAT), *Tgfβ* (forward: GACCGCAACAACGCCATC and reverse: CACTGCTTCCCGAATGTCTGA), *αSMA* (forward: GACTACTGCCGAGCGTGAG and reverse: CCGTCAGGCAGTTCGTAGC), *Mmp2* (forward: TCACTTTCCTGGGCAACAAGT and reverse: GCCACGAGGAATAGGCTATATCC) and *Timp2* (forward: GATTCAGTATGAGATCAAGCAGATAAAGA and reverse: GCGAGACCCCGCACACT). All samples were run in duplicate using the QuantStudio 7 Flex Real-Time PCR system (ThermoFisher; Massachusetts, USA).

### Immunohistochemistry

Four-micron thick paraffin sections of kidneys were deparaffinised, rehydrated and rinsed. Heat-mediated antigen retrieval method with sodium citrate buffer (10 mM sodium citrate, 0.05% tween 20, pH 6.0) was performed. Sections were then quenched with 3% H_2_0_2_ for 20 minutes and incubated with 10% (rabbit/goat) serum for 30 minutes to block non-specific binding. Sections were incubated with rat monoclonal to IL-10 (ab189392; Abcam, Cambridge, UK) or rabbit polyclonal to PAI-1 (ab66705; Abcam, Cambridge, UK) antibody (1:100) overnight at 4 °C. The next day, sections were washed and incubated with anti-rat/anti-rabbit secondary antibody (1:200) for 30 minutes at room temperature. This was followed by incubation with horseradish peroxidase-conjugated streptavidin (VECTASTAIN Elite ABC staining kit; Vector Laboratories). 3,3′-diaminobenzidine tetrahydrochloride was then used to visualise peroxidase conjugates in samples. All slides were then hydrated, cleared and mounted with DPX. Ten random fields per animal were imaged in the renal cortical region using the Olympus BX43 microscope (x20 magnification). Staining was quantified by counting the number of positively stained cells per field. All assessments were performed in a blinded manner.

### Hydroxyproline assay

Hydroxyproline assay was performed as previously described^51^.

### Echocardiography data analysis

Images obtained from echocardiography were analysed to measure interventricular septum thickness during diastole and systole (IVSd/s), left ventricular posterior wall thickness during diastole and systole (LVPWd/s), left ventricular end-diastolic/end-systolic dimension (LVDD/LVSD) and heart rate. Fractional shortening (FS) was then calculated using the following formula: [(LVDD-LVSD)/LVDD] × 100%.

### Statistics

GraphPad Prism (Version 6; GraphPad Software; San Diego, USA) was used to perform all statistical analyses. Data are shown as mean ± SEM. One-way ANOVA followed by Tukey post-hoc tests for multiple comparisons was used to compare the effects of treatments. Unpaired t-tests were used for dichotomous comparisons. Two-tailed *P* ≤ 0.05 was considered statistically significant.

## Electronic supplementary material

Supplementary Dataset 1
