# Supplementary material for: N-Acetylcysteine Attenuates the Development of Renal Fibrosis in Transgenic Mice with Dilated Cardiomyopathy
Source: Sci Rep. 2017 Dec 18;7:17718. doi: 10.1038/s41598-017-17927-5 (PMC5735149; doi:10.1038/s41598-017-17927-5)
Supplement: Supplementary file 1 — Supplementary Dataset 1 [file 41598_2017_17927_MOESM1_ESM.pdf]

N-Acetylcysteine Attenuates the Development of Renal Fibrosis in Transgenic Mice with Dilated Cardiomyopathy

Beverly Giam, BSc(Hons)[1,2]*, Sanjaya Kuruppu, PhD[3], Po-Yin Chu, PhD[1], A. Ian Smith, PhD[3], Francine Z. Marques, PhD[1,2], April Fiedler, BSc(Hons)[1], Duncan Horlock, BSc(Hons)[1], Helen Kiriazis, PhD[1], Xiao-Jun Du, MD, PhD[1], David M. Kaye, MD, PhD[1,4]+ and Niwanthi W. Rajapakse, PhD[1,5]+

[1]Baker Heart and Diabetes Institute, Melbourne, Australia, [2]Central Clinical School, Monash University, Melbourne, Australia, [3]Biomedicine Discovery Institute, Department of Biochemistry & Molecular Biology, Monash University, Melbourne, Australia. [4]Department of Medicine, Monash University, Melbourne, Australia. [5]School of Biomedical Sciences, Faculty of Medicine, University of Queensland, Brisbane, Australia.

+These authors contributed equally to this work

Corresponding author:
Miss Beverly Giam,
Heart Failure Research Group,
Baker Heart and Diabetes Institute,
75, Commercial Road, Melbourne, 3004,
Australia.
Phone: 613 8532 1919
Fax: 613 8532 1100
E-mail: beverly.giam@baker.edu.au

Supplementary figure S1

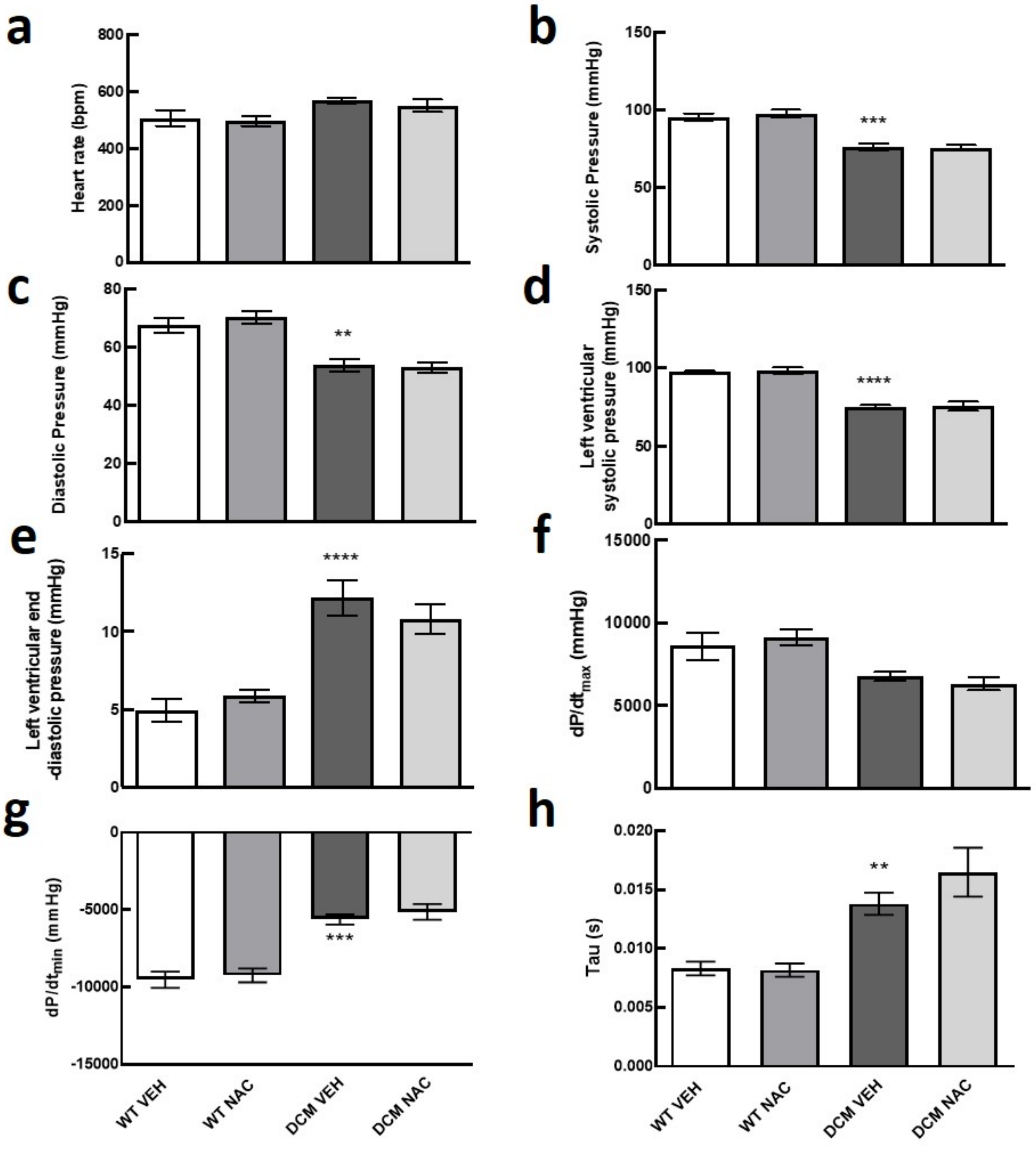

Supplementary fig. S1 **a:** Heart rate, **b-c:** aortic, **d-e:** ventricular pressure, **f:** dP/dt_{max} ,**g:** dP/dt_{min} and **h:** Tau in WT and DCM mice administered NAC or saline (n=4-8). Data are mean ± SEM. **P<0.01, ***P<0.001 *vs* saline treated WT mice. P values were derived from a one-way ANOVA followed by Tukey post-hoc test. dP/dt_{max} = maxmimal rate of increase in left ventricular pressure, dP/dt_{min} = minimal rate of decay in left ventricular pressure. Other abbreviations are as for fig. 1.

Supplementary figure S2

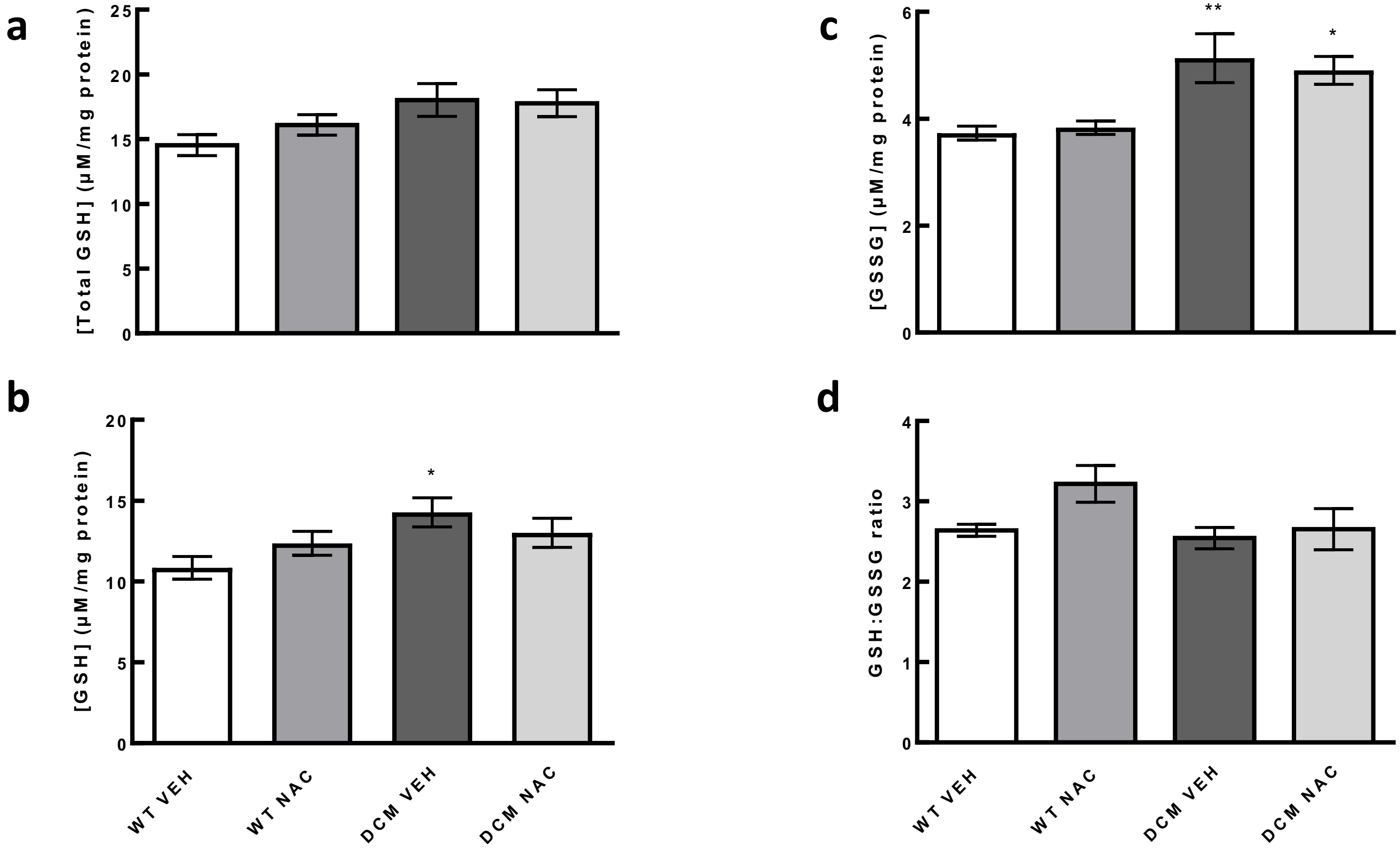

Supplementary fig. S2 Levels of glutathione after 8 weeks of NAC or saline treatments (n=5-6). Data are mean ± SEM. **P*≤0.05, ***P*≤0.01 vs saline treated WT mice. *P* values were derived from a one-way ANOVA followed by Tukey post-hoc test. Total GSH = total glutathione content, GSSG = oxidised glutathione content, GSH = reduced glutathione content, GSH:GSSG = reduced glutathione: oxidised glutathione ratio. Other abbreviations are as for fig. 1.

Supplementary figure S3

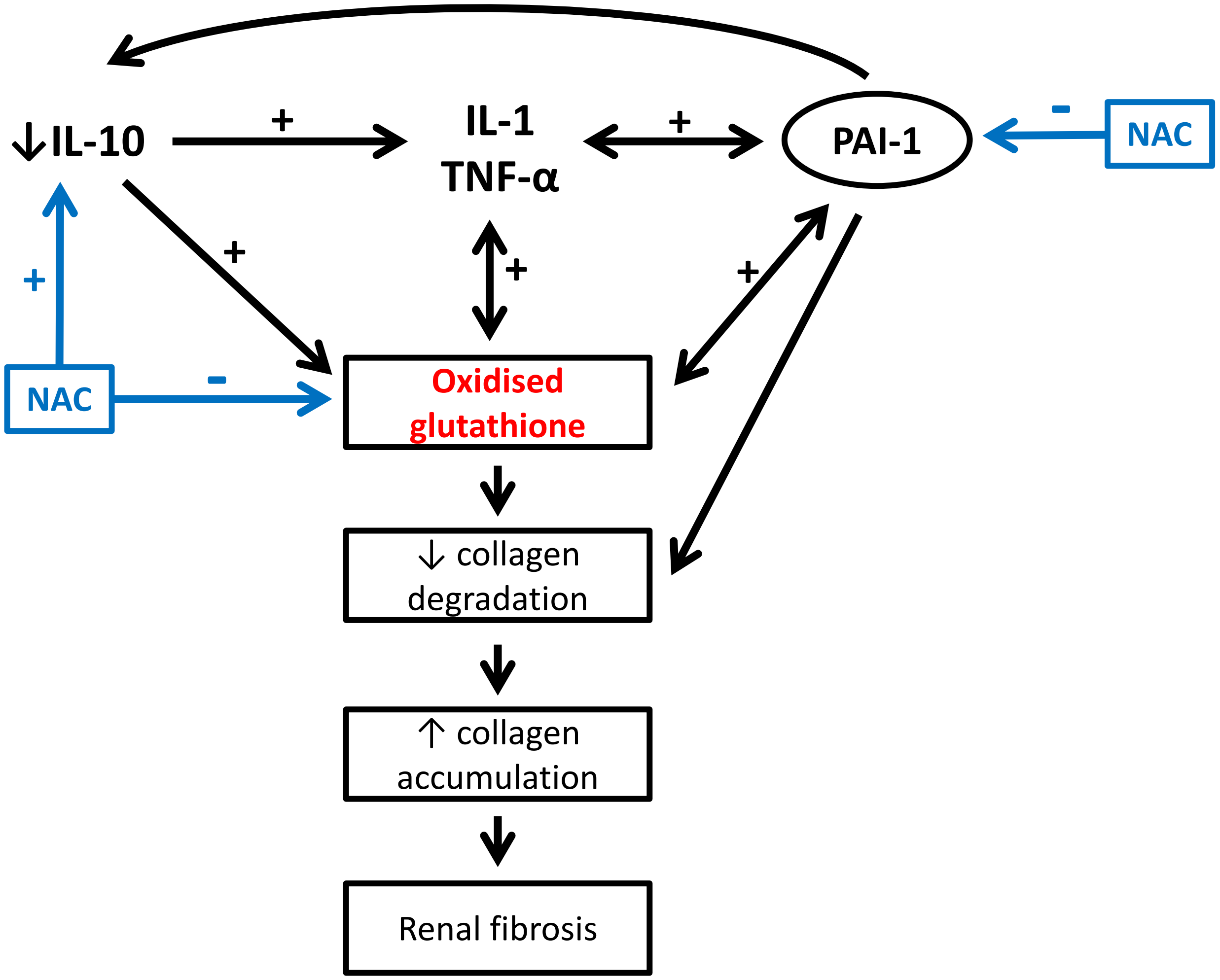

Supplementary fig. S3 Renal inflammation is present in dilated cardiomyopathy. This can augment the levels of oxidised glutathione, which in turn, can reduce collagen degradation. This leads to fibrosis. Glutathione precursor, n-acetylcysteine, can normalise glutathione levels, inhibit PAI-1 expression and increase expression of anti-inflammatory cytokine, IL-10. This can reduce the formation of fibrosis and potentially reverse established fibrosis. IL-10 = interleukin 10, IL-1 = interleukin 1, TNF-α = tumour necrosis factor α, PAI-1 = plasminogen activator inhibitor 1, NAC = n-acetylcysteine.

Supplementary table S1: Cardiac function at baseline and during NAC or saline treatment

	WT Vehicle		WT NAC		DCM Vehicle		DCM NAC	
	**Baseline**	**Saline**	**Baseline**	**NAC**	**Baseline**	**Saline**	**Baseline**	**NAC**
**Group size**	**9**	**9**	**9**	**9**	**9**	**9**	**7**	**7**
FS, %	32±3	35±2	36±2	32±3	18±1***	15±1***	17±2***	17±3***

Values are means ± SEM. ***P<0.001 vs age matched saline treated WT mice. *P* values were derived from a one-way ANOVA followed by Tukey post-hoc test. WT= wild type, DCM = transgenic mice with dilated cardiomyopathy, NAC = n-acetylcysteine, FS = fractional shortening.

Supplementary table S2: Echocardiographic features of WT and DCM mice at baseline

	WT Vehicle	WT NAC	DCM Vehicle	DCM NAC
**Group size**	**9**	**9**	**9**	**7**
IVSd, mm	0.77±0.03	0.73±0.02	0.50±0.03***	0.53±0.04**
IVSs, mm	1.35±0.03	1.32±0.06	0.76±0.06***	0.77±0.08***
LVPWd, mm	0.79±0.01	0.78±0.02	0.71±0.04	0.66±0.02*
LVPWs, mm	1.20±0.06	1.16±0.06	0.90±0.04***	0.82±0.02***
LVDD, mm	4.13±0.11	4.01±0.13	4.38±0.10	4.67±0.11*
LVSD, mm	2.75±0.13	2.53±0.16	3.60±0.08***	3.94±0.19***
HR, bpm	552±15	592±56	511±29	503±18

Values are means ± SEM. **P*<0.05, ***P*<0.01, ****P*<0.001 *vs* saline treated WT mice. *P* values were derived from a one-way ANOVA followed by Tukey post-hoc test. IVS = interventricular septum (anterior wall), LVPW = left ventricular posterior wall, LVDD = LV end-diastolic dimension, LVSD = LV end-systolic dimension, HR = heart rate. Other abbreviations are as for supplementary table S1.

Supplementary table S3: Echocardiographic features of WT and DCM mice treated with saline or NAC

	WT Vehicle	WT NAC	DCM Vehicle	DCM NAC
**Group size**	**9**	**9**	**9**	**7**
IVSd, mm	0.88±0.05	0.80±0.04	0.60±0.02***	0.70±0.07*
IVSs, mm	1.39±0.06	1.33±0.06	0.85±0.04***	0.94±0.13***
LVPWd, mm	0.88±0.02	0.80±0.03	0.73±0.03**	0.82±0.05
LVPWs, mm	1.36±0.05	1.18±0.07	0.88 ±0.04***	0.98±0.05*
LVDD, mm	4.01 ±0.12	4.08±0.10	4.49±0.14	4.50±0.23
LVSD, mm	2.60±0.18	2.69±0.13	3.86±0.14***	3.78±0.29***
HR, bpm	549 ±12	526±17	480±22	549±25

Values are means ± SEM. **P*<0.05, ***P*<0.01, ****P*<.0001 *vs* saline treated WT mice. *P* values were derived from a one-way ANOVA followed by Tukey post-hoc test. Abbreviations are as for supplementary table S2.

Supplementary table S4: Body weight and lung weight

	WT Vehicle	WT NAC	DCM Vehicle	DCM NAC
**Group size**	**6**	**6**	**6**	**6**
Body weight, g	33.0±0.5	32.0±0.5	33.0±0.6	32.0±0.6
Lung weight, mg	192±9	179±8	245±13**	235±11

Values are means ± SEM. ***P*<0.01 *vs* saline treated WT mice. *P* values were derived from a one-way ANOVA followed by Tukey post-hoc test. Abbreviations are as for supplementary table S1.
